# Toward Shared Autonomy Control Schemes for Human-Robot Systems: Action Primitive Recognition Using Eye Gaze Features

**DOI:** 10.3389/fnbot.2020.567571

**Published:** 2020-10-15

**Authors:** Xiaoyu Wang, Alireza Haji Fathaliyan, Veronica J. Santos

**Affiliations:** Biomechatronics Laboratory, Mechanical and Aerospace Engineering, University of California, Los Angeles, Los Angeles, CA, United States

**Keywords:** action primitive recognition, activities of daily living, eye gaze, gaze-object angle, human-robot systems, recurrent neural network, shared autonomy

## Abstract

The functional independence of individuals with upper limb impairment could be enhanced by teleoperated robots that can assist with activities of daily living. However, robot control is not always intuitive for the operator. In this work, eye gaze was leveraged as a natural way to infer human intent and advance action recognition for shared autonomy control schemes. We introduced a classifier structure for recognizing low-level action primitives that incorporates novel three-dimensional gaze-related features. We defined an action primitive as a triplet comprised of a verb, target object, and hand object. A recurrent neural network was trained to recognize a verb and target object, and was tested on three different activities. For a representative activity (making a powdered drink), the average recognition accuracy was 77% for the verb and 83% for the target object. Using a non-specific approach to classifying and indexing objects in the workspace, we observed a modest level of generalizability of the action primitive classifier across activities, including those for which the classifier was not trained. The novel input features of gaze object angle and its rate of change were especially useful for accurately recognizing action primitives and reducing the observational latency of the classifier.

## Introduction

Activities of daily living (ADLs) can be challenging for individuals with upper limb impairment. The use of assistive robotic arms is an active area of research, with the aim of increasing an individual's functional independence (Groothuis et al., 2013). However, current assistive robotic arms, such as the Kinova arm and Manus arm, are controlled by joysticks that require operators to frequently switch between several modes for the gripper, including a position mode, an orientation mode, and an open/close mode (Driessen et al., 2001; Maheu et al., 2011). Users need to operate the arm from the gripper's perspective, in an unintuitive Cartesian coordinate space. Operators would greatly benefit from a control interface with a lower cognitive burden that can accurately and robustly inference human intent.

The long-term objective of this work is to advance shared autonomy control schemes so that individuals with upper limb impairment can more naturally control robots that assist with activities of daily living. Toward this end, the short-term goal of this study is to advance the use of eye gaze for action recognition. Our approach is to develop a neural-network based algorithm that exploits eye gaze-based information to recognize action primitives that could be used as modular, generalizable building blocks for more complex behaviors. We define new gaze-based features and show that they increase recognition accuracy and decrease the observational latency (Ellis et al., 2013) of the classifier.

This article is organized as follows. Section Related Work outlines related work with respect to user interfaces for assistive robot arms and action recognition methods. Section Materials and Methods introduces the experimental protocol and proposed structure of an action primitive recognition model, whose performance is detailed in section Results. Section Discussion addresses the effects of input features on classifier performance and considerations for future real-time implementation. Contributions are summarized in section Conclusion.

## Related Work

### User Interfaces for Assistive Robot Arms

Many types of non-verbal user interfaces have been developed for controlling assistive robot arms that rely on a variety of input signals, such as electrocorticographic (ECoG) (Hochberg et al., 2012), gestures (Rogalla et al., 2002), electromyography (EMG) (Bi et al., 2019), and electroencephalography (EEG) (Bi et al., 2013; Salazar-Gomez et al., 2017). Although ECoG has been mapped to continuous, high-DOF hand and arm motion (Chao et al., 2010; Wang et al., 2013), a disadvantage is that an invasive surgical procedure is required. Gesture-based interfaces often require that operators memorize mappings from specific hand postures to robot behaviors (Rogalla et al., 2002; Ghobadi et al., 2008; Raheja et al., 2010), which is not natural. EMG and EEG-based interfaces, although non-invasive and intuitive, require users to don and doff EMG electrodes or an EEG cap, which may be inconvenient and require a daily recalibration.

In this work, we consider eye gaze-based interfaces, which offer a number of advantages. Eye gaze is relatively easy to measure and can be incorporated into a user interface that is non-verbal, non-invasive, and intuitive. In addition, with this type of interface, it may be possible to recognize an operator's intent in advance, as gaze typically precedes hand motions (Hayhoe et al., 2003).

Numerous studies have reported on the use of eye gaze for robot control. In the early 2000's, the eyetracker was used as a *direct substitute* for a handheld mouse such that the gaze point on a computer display designates the cursor's position, and blinks function as button clicks (Lin et al., 2006; Gajwani and Chhabria, 2010). Since 2015, eye gaze has been used to communicate a 3D *target position* (Li et al., 2015a, 2017; Dziemian et al., 2016; Li and Zhang, 2017; Wang et al., 2018; Zeng et al., 2020) for directing the movement of the robotic end effector. No action recognition was required, as these methods assumed specific actions in advance, such as reach and grasp (Li et al., 2017), write and draw (Dziemian et al., 2016), and pick and place (Wang et al., 2018). Recently, eye gaze has been used to *recognize an action* from an a priori list. For instance, Shafti et al. developed an assistive robotic system that recognized subjects' intended actions (including reach to grasp, reach to drop, and reach to pour) using a finite state machine (Shafti et al., 2019).

In this work, we advance the use of eye gaze for action recognition. We believe that eye gaze control of robots is promising due to the non-verbal nature of the interface, the rich information that can be extracted from eye gaze, and the low cognitive burden on the operator during tracking of natural eye movements.

### Action Representation and Recognition

Moeslund et al. described human behaviors as a composition of three hierarchical levels: (i) activities, (ii) actions, and (iii) action primitives (Moeslund et al., 2006). At the highest level, activities involve a number of actions and interactions with objects. In turn, each action is comprised of a set of action primitives. For example, the activity “making a cup of tea” is comprised of a series of actions, such as “move the kettle to the stove.” This specific action can be further divided into three action primitives: “dominant hand reaches for the kettle,” “dominant hand moves the kettle to the stove,” and “dominant hand sets down the kettle onto the stove.”

A great body of computer vision-based studies has already contributed to the recognition of activities of daily living such as walk, run, wave, eat, and drink (Lv and Nevatia, 2006; Wang et al., 2012; Vemulapalli et al., 2014; Du et al., 2015). These studies detected joint locations and joint angles as input features from external RGB-D cameras and classified ADLs using algorithms such as hidden Markov models (HMMs) and recurrent neural networks (RNNs).

Other studies leveraged egocentric videos taken by head-mounted cameras or eyetrackers (Yu and Ballard, 2002; Yi and Ballard, 2009; Fathi et al., 2011, 2012; Behera et al., 2012; Fathi and Rehg, 2013; Matsuo et al., 2014; Li et al., 2015b; Ma et al., 2016). Video preprocessing methods necessitated first subtracting the foreground and then detecting human hands and activity-relevant objects. Multiple features related to hands, objects, and gaze were then used as inputs for the action recognition using approaches such as HMMs, neural networks, and support vector machines (SVMs). Hand-related features included hand pose, hand location, relationship between left and right hand, and the optical flow field associated with the hand (Fathi et al., 2011; Ma et al., 2016). Object-related features included pairwise spatial relationships between objects (Behera et al., 2012), state changes of an object (open vs. closed) (Fathi and Rehg, 2013), and the optical flow field associated with objects (Fathi et al., 2011). The “visually regarded object,” defined by Yi and Ballard (2009) as the object being fixated by the eyes, was widely used as the gaze-related feature (Yu and Ballard, 2002; Yi and Ballard, 2009; Matsuo et al., 2014). Some studies additionally extracted features such as color and texture near the visually regarded object (Fathi et al., 2012; Li et al., 2015b).

Due to several limitations, state-of-the-art action recognition methods cannot be directly applied to the intuitive control of an assistive robot via eye gaze. First, computer vision-based approaches to the automated recognition of ADLs have focused on the activity and action levels according to Moeslund's description of action hierarchy (Moeslund et al., 2006). Yet, state-of-the-art robots are not sophisticated enough to autonomously plan and perform these high-level behaviors. Second, eye movements are traditionally used to estimate gaze point or gaze object alone (Yu and Ballard, 2002; Yi and Ballard, 2009; Matsuo et al., 2014). More work could be done to extract other useful features from spatiotemporal eye gaze data, such as time histories of gaze object angle and gaze object angular speed, which are further described in section Gaze-Related Quantities.

## Materials and Methods

### Experimental Set-Up

**Figure 1:**
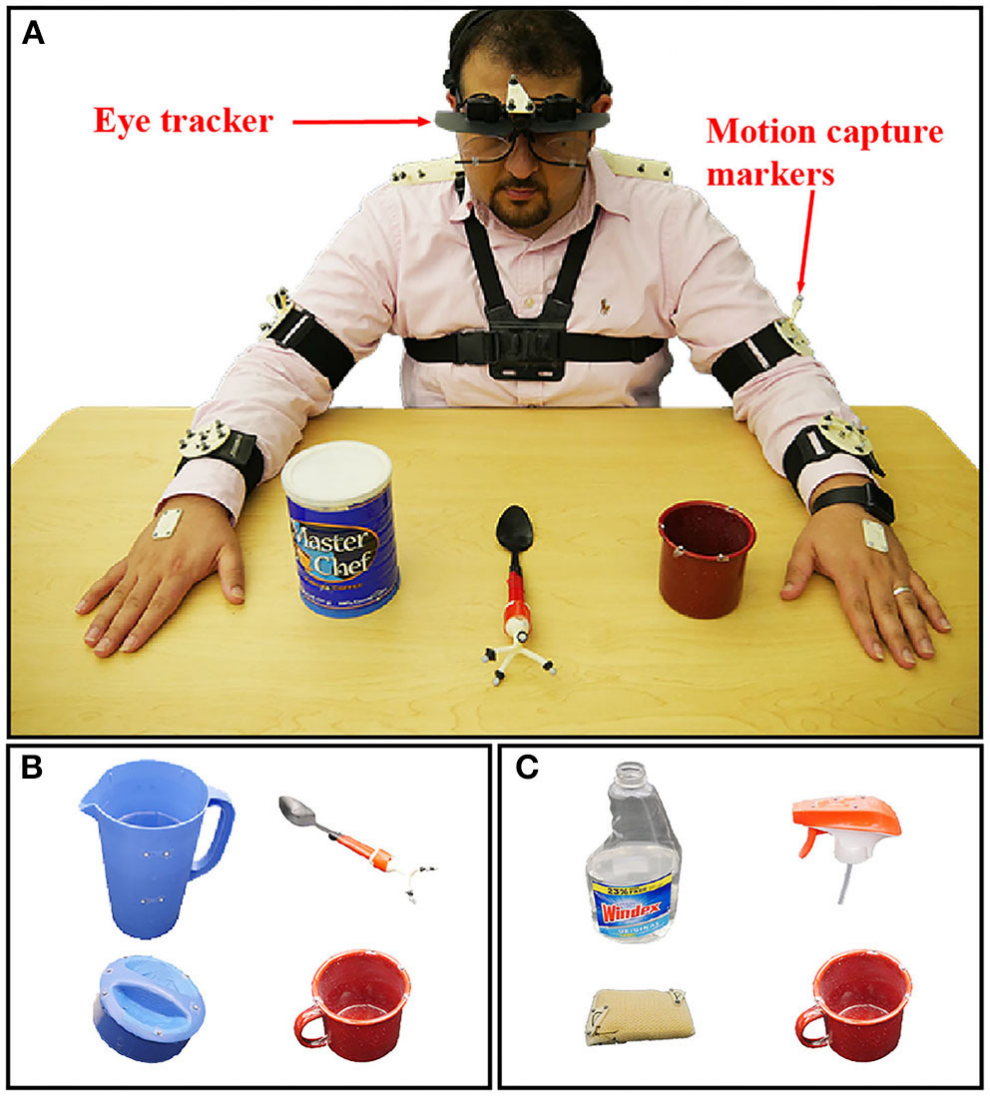
**(A)** A subject prepares to perform Activity 2 (make instant coffee) while eye gaze and kinematics are tracked with a head-mounted eyetracker and motion capture system (not shown). Activity 2 involves a coffee can, spoon and mug. **(B)** Activity 1 (make a powdered drink) involves a coffee can, spoon and mug. **(C)** Activity 3 (prepare a cleaning sponge) involves a spray bottle and cap, sponge, and mug. The subject shown in panel **(A)** has approved of the publication of this image.

This study was approved by the UCLA Institutional Review Board. The experimental setup and protocol were previously reported in our prior paper (Haji Fathaliyan et al., 2018). Data from 10 subjects are reported [nine males, one female; aged 18–28 years; two pure right-handers, six mixed right-handers, two neutral, per a handedness assessment (Zhang, 2012) based on the Edinburgh Handedness Inventory (Oldfield, 1971)]. Subjects were instructed to perform three bimanual activities involving everyday objects and actions: make instant coffee, make a powdered drink, and prepare a cleaning sponge (Figure 1). The objects involved in these three activities were selected from the benchmark Yale-CMU-Berkeley (YCB) Object Set (Calli et al., 2015). We refer to these objects as *activity-relevant objects* since they would be grasped and manipulated as subjects performed specific activities.

For Activity 1, subjects removed a pitcher lid, stirred the water in the pitcher, and transferred the water to a mug using two different methods (scooping with a spoon and pouring). For Activity 2, subjects were instructed to remove a coffee can lid, scoop instant coffee mix into a mug, and pour water from a pitcher into the mug. For Activity 3, subjects unscrewed a spray bottle cap, poured water from the bottle into a mug, sprayed the water onto a sponge, and screwed the cap back onto the bottle. In order to standardize the instructions provided to subjects, the experimental procedures were demonstrated via a prerecorded video. Each activity was repeated by the subject four times; the experimental setup was reset prior to each new trial.

A head-mounted eyetracker (ETL-500, ISCAN, Inc., Woburn, MA, USA) was used to track the subject's gaze point at 60 Hz with respect to a built-in egocentric scene camera. Per calibration data, the accuracy and precision of the eyetracker were ~1.4 deg and 0.1 deg, respectively. The motion of the YCB objects, eyetracker, and each subject's upper limb were tracked at 100 Hz by six motion capture cameras (T-Series, Vicon, Culver City, CA, USA). A blackout curtain surrounded the subject's field of view in order to minimize visual distractions. A representative experimental trial is shown in Supplementary Video 1.

### Gaze-Related Quantities

We extract four types of gaze-related quantities from natural eye movements as subjects performed Activities 1–3. The quantities include the *gaze object* (GO) (Yu and Ballard, 2002; Yi and Ballard, 2009; Matsuo et al., 2014) and *gaze object sequence* (GOS) (Haji Fathaliyan et al., 2018). This section describes how these quantities are defined and constructed. As described in section Input Features for the Action Primitive Recognition Model, these gaze-related quantities are used as inputs to a long-short term memory (LSTM) recurrent neural network in order to recognize action primitives.

The raw data we obtain from the eyetracker is a set of 2D pixel coordinates. The coordinates represent the perspective projection of a subject's gaze point onto the image plane of the eyetracker's egocentric scene camera. In order to convert the 2D pixel coordinate into a 3D gaze vector, we use camera calibration parameters determined using a traditional chessboard calibration procedure (Heikkila and Silven, 1997) and the MATLAB Camera Calibration Toolbox (Bouguet, 2015). The 3D gaze vector is constructed by connecting the origin of the egocentric camera frame with the gaze point location in the 2D image plane that is now expressed in the 3D global reference frame.

The *gaze object (GO)* is defined as the first object to be intersected by the 3D gaze vector, as the gaze vector emanates from the subject. Thus, if the gaze vector pierces numerous objects, then the object that is closest to the origin of the 3D gaze vector (within the head-mounted eyetracker) is labeled as the gaze object.

**Figure 2:**
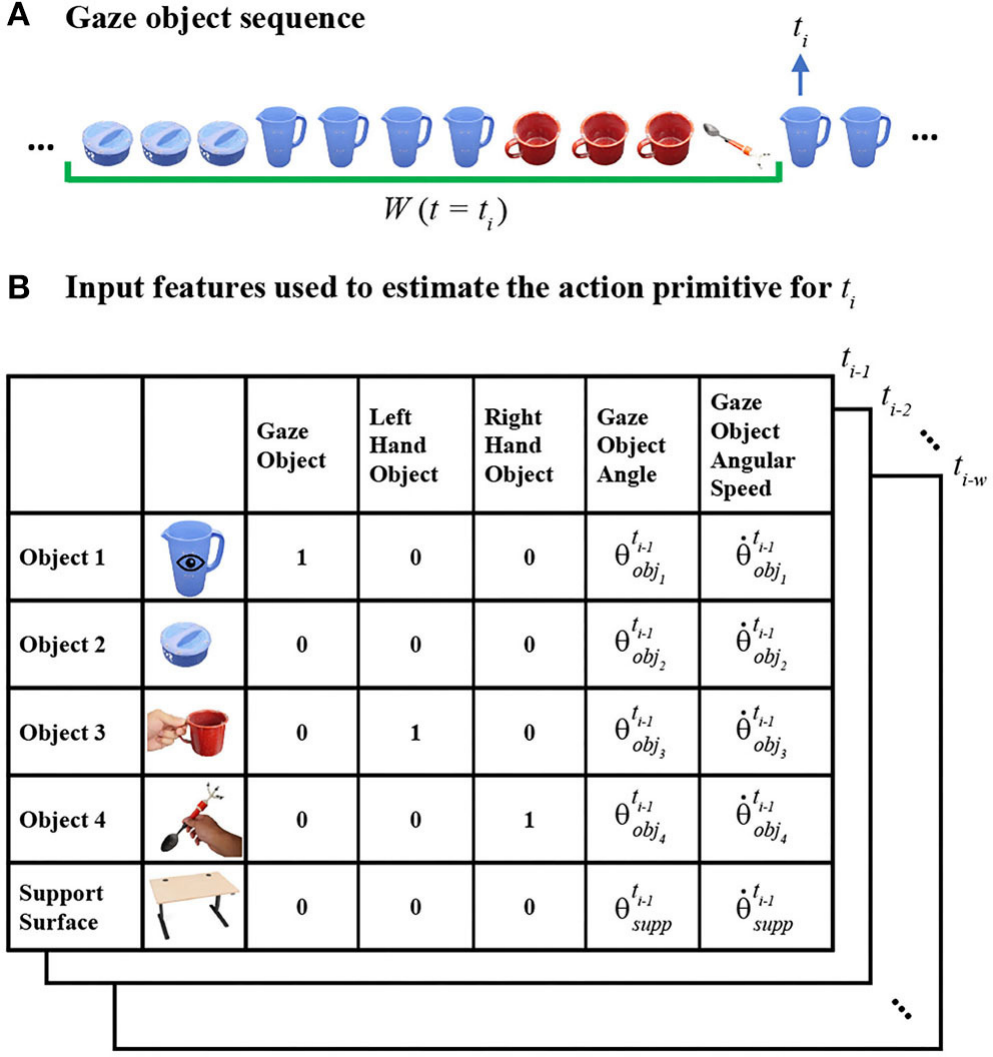
**(A)** The gaze object sequence time history GOS(t_i_) within a window of time W(t_i_) (green bracket) is shown for Activity 1 (make a powdered drink). **(B)** To predict the action primitive at time step t_i_, input feature vectors (shown as 5 ×5 matrices for clarity) are created for each of the times from t_i−w_ to t_i−1_. Activity-relevant objects are sorted according to their frequency of occurrence in the GOS(t_i_).

As defined in our prior paper, the *gaze object sequence (GOS)* refers to the identity of the gaze objects in concert with the sequence in which the gaze objects are visually regarded (Haji Fathaliyan et al., 2018). Specifically, the gaze object sequence time history *GOS(t*_*i*_*)* is comprised of a sequence of gaze objects sampled at 60 Hz within a given window of time *W(t*_*i*_*)* (Figure 2). The time window *W(t*_*i*_*)* contains *w* time steps from *t*_*i*−*w*_ to *t*_*i*−1_.

In this work, we use a value of *w* = 75 time steps, equivalent to 1.25 s. This time window size was determined from a pilot study whose results are presented in section Effect of Time Window Size on Recognition Accuracy. The pilot study was motivated by the work of Haseeb et al. in which the accuracy of an LSTM RNN was affected by time window size (Haseeb and Parasuraman, 2017).

**Figure 3:**
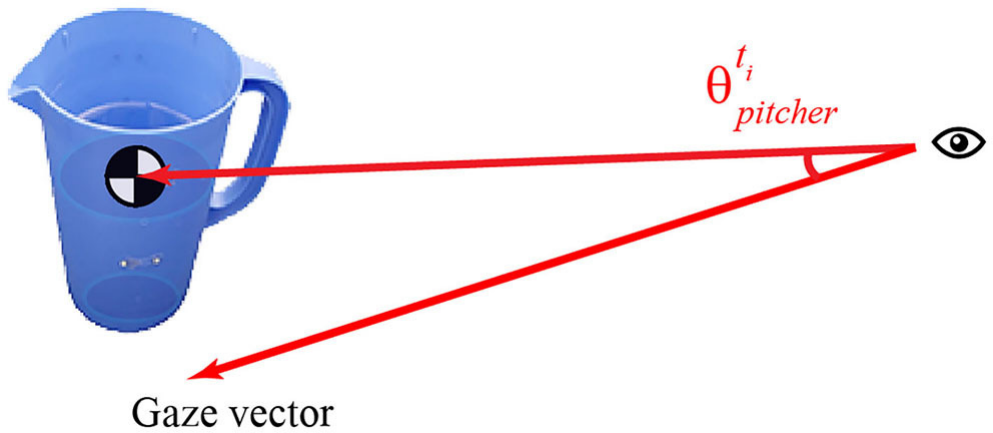
Gaze object angle is defined as the angle between the gaze vector and the eye-object vector (ending at the object's center of mass).

The *gaze object angle* (GOA) describes the spatial relationship between the gaze vector and each gaze object. The GOA is defined as the angle between the gaze vector and the eye-object vector (Figure 3). The eye-object vector shares the same origin as the gaze vector but ends at an object's center of mass. Each object's center of mass was estimated by averaging the 3D coordinates of the points in the object's point cloud. Each object's point cloud was scanned with a structured-light 3D scanner (Structure Sensor, Occipital, Inc., CA, USA) and custom turntable apparatus. Containers, such as the pitcher and mug, are assumed to be empty for center of mass estimation.

The *gaze object angular speed* (GOAS) is calculated by taking the time derivative of the GOA. We use the GOAS to measure how the gaze vector moves with respect to other activity-relevant objects. Previously, the gaze object and gaze object sequence have been used to recognize actions (Yi and Ballard, 2009; Matsuo et al., 2014). To our knowledge, this is the first work to leverage the gaze object angle and gaze object angular speed for action primitive recognition.

### Action Primitive Recognition Model

#### Action Primitive Representation

We represent each action primitive as a triplet comprised of a *verb, target object (TO)*, and *hand object (HO)*. Each action primitive can be performed by either the dominant hand or non-dominant hand. When both hands are active at the same time, hand-specific action primitives can occur concurrently.

The verb can be one of four classes: *Reach, Move, Set down, or Manipulate*. The classes Reach, Move, and Set down describe hand movements toward an object or support surface, with or without an object in the hand. Notably, these verbs are not related to or dependent upon object identity. In contrast, the class Manipulate includes a list of verbs that are highly related to object-specific affordances (Gibson, 1977). For instance, in Activity 1, the verb “scoop” and “stir” are closely associated with the object “spoon” (Table 1). We refer to these verbs as *manipulate-type verbs*.

**Table 1:** Each of three activities is divided into actions that are further decomposed into action primitives. Each action primitive is defined as a triplet comprised of a verb, target object (TO), and hand object (HO).

**Activities**		**Activity 1: make a powdered drink**	**Activity 2: make instant coffee**	**Activity 3: prepare a cleaning sponge**
**Actions**		Remove pitcher lid Stir liquid inside pitcher Scoop liquid into mug Close pitcher lid Pour liquid into mug	Remove coffee can lid Scoop coffee insider can Transfer coffee into mug Stir liquid inside mug Close coffee can lid	Remove spray bottle cap Transfer cleanser into mug Close spray bottle cap Spray cleanser onto sponge
**Action primitives**	**Verb**	Reach, Move, Set down, Manipulate (open, close, stir, scoop, drop, pour)	Reach, Move, Set down, Manipulate (open, close, stir, scoop, drop, pour)	Reach, Move, Set down, Manipulate (screw, unscrew, lift, pour, insert, spray)
	**TO**	Pitcher, pitcher lid, mug, spoon, table	Coffee can, coffee lid, mug, spoon, table	Spray bottle, spray cap, mug, sponge, table
	**HO**	Pitcher, pitcher lid, mug, spoon	Coffee can, coffee lid, mug, spoon	Spray bottle, spray cap, mug, sponge

In addition to a verb, the action primitive triplet includes the identity of two objects. The *target object* TO refers to the object that will be directly affected by verbs such as Reach, Move, Set down, and Manipulate. The *hand object* HO refers to the object that is currently grasped. For instance, when the dominant hand grasps a spoon and stirs inside a mug, the triplet of the action primitive for the dominant hand is: manipulate (verb), mug (TO), and spoon (HO). A hierarchical description of activities, actions, and action primitives for Activities 1–3 are presented in Table 1.

In order to develop a supervised machine learning model for action primitive recognition, we manually label each time step with the action primitive triplet for either the dominant or non-dominant hand. The label is annotated using video recorded by an egocentric scene camera mounted on the head-worn eyetracker. We annotate each time step with the triplet of a subject's dominant hand as it is more likely the target of the subject's attention. For instance, when the dominant hand (holding a spoon) and the non-dominant hand (holding a mug) move toward each other simultaneously, we label the action primitive as “move the spoon to the mug,” where the verb is “move” and the target object is “mug.” However, when the dominant hand is not performing any action primitive, we refer to the non-dominant hand instead. If neither hand is moving or manipulating an object, we exclude that time step from the RNN training process.

#### Input Features for the Action Primitive Recognition Model

Given that the identity of gaze objects will vary across activities, we substitute the specific identities of gaze objects with numerical indices. This is intended to improve the generalizability of our action primitive recognition algorithm across different activities. For each time step *t*_*i*_, the *n* activity-relevant objects are sorted in descending order according to their frequency of occurrence in *GOS(t*_*i*_*)*. Once sorted, the objects are indexed as Object 1 to Object *n*, such that Object 1 is the object that most frequently appears in the gaze object sequence at *t*_*i*_. If two or more objects appear in the gaze object sequence with the same frequency, the object with the smaller gaze object angle is assigned the smaller numerical index, as it is aligned most closely to the gaze vector and will be treated preferentially.

Figure 2 exemplifies how activity-relevant objects in a gaze object sequence would be assigned indices at a specific time step *t*_*i*_. The activity-relevant objects (*n* = 4) in Activity 1 were sorted according to their frequency of occurrence in *GOS(t*_*i*_*)*, which is underlined by a green bracket in Figure 2A. Based on frequency of occurrence, the activity-relevant objects were indexed as follows: pitcher (Object 1), pitcher lid (Object 2), mug (Object 3), and spoon (Object 4).

We introduce here the idea of a “support surface,” which could be a table, cupboard shelf, etc. In this work, we do not consider the support surface (experiment table) as an activity-relevant object, as it cannot be moved or manipulated and does not directly affect the performance of the activity. Nonetheless, the support surface still plays a key role in the action primitive recognition algorithm due to the strong connection with the verb Set down. In addition, the support surface frequently appears in the *GOS*.

To predict the action primitive at time step t_i_, input feature vectors are created for each of the time steps from time t_i−w_ to t_i−1_, as shown in Figure 2B. For Activity 1, each input feature vector consists of five features for each of four activity-relevant objects and a support surface. For clarity, each resulting 25 ×1 feature vector is shown as a five-by-five matrix in Figure 2B. Gaze object, left-hand object, and right-hand object are encoded in the form of one-hot vectors while gaze object angle and angular speed are scalar values.

Gaze object identity was included as an input feature because it supported action recognition in prior studies (Yu and Ballard, 2002; Yi and Ballard, 2009; Matsuo et al., 2014). We included the hand object as an input feature although it is a component of the action primitive triplet that we seek to recognize. Considering the application of controlling a robotic arm through eye gaze, we expect the robotic system to determine an object's identity before it plans any movements with respect to the object. As a result, we assume that the hand object's identity is always accessible to the classification algorithm. We included the GOA and GOAS as input features because we hypothesized that spatiotemporal relationships between eye gaze and objects would be useful for action primitive recognition. The preprocessing pipeline for the input features is shown in Supplementary Video 1.

#### Action Primitive Recognition Model Architecture

We train a long short-term memory (LSTM) recurrent neural network to recognize the verb and the target object *TO* for each time step *t*_*i*_. With this supervised learning method, we take as inputs the feature vectors described in section Input Features for the Action Primitive Recognition Model. For the RNN output, we label each time step *t*_*i*_ with a pair of elements from a discrete set of verbs and generic, indexed target objects:

(1) $$\begin{array}{r} \text{Verb}\left(t_{i}\right)\in V=\left\{Reach,\;Move,Set\;down,\;Manipulate\right\} \end{array}$$

(2) $$\begin{array}{r} \text{TO}\left(t_{i}\right)\in O=\left\{Object_{1},\;Object_{2},Object_{3},Support\;surface\right\} \end{array}$$

The target object class Object 4 was excluded from the model output since its usage accounted for <1% of the entire dataset. The four verb labels and four TO labels are combined as 16 distinct verb-TO pairs, which are then taken as output classes when we train the RNN.

(3) $$\begin{array}{l} \left(Verb\left(t_{i}\right),TO\left(t_{i}\right)\right)\in O\times V\\ =\left\{\;\left(Reach,Object_{1}\right),\ldots ,\left(Manipulate,Support\;surface\right)\;\right\} \end{array}$$

As a result, verb-TO pairs that never occur during the training process, such as (Manipulate, Support surface), can be easily eliminated.

In order to evaluate the RNN's performance on the verb and target object individually, we split the verb-TO pairs after recognition. A softmax layer was used as the final layer of the RNN.

(4) $$\begin{array}{r} Verb\left(t_{i}\right)=argmax_{\in V}\left(\underset{\in O}{\sum }softmax\left(\left(Verb\left(t_{i}\right)=,TO\left(t_{i}\right)=\right)\right)\right) \end{array}$$

(5) $$\begin{array}{r} TO\left(t_{i}\right)=\;argmax_{\in O}\left(\underset{\in V}{\sum }softmax\left(\left(Verb\left(t_{i}\right)=,TO\left(t_{i}\right)=\right)\right)\right) \end{array}$$

The RNN was comprised of one LSTM layer, three dense layers, and one softmax layer. The LSTM contained 64 neurons and each of the three dense layers contained 30 neurons. The RNN was trained with an Adaptive Momentum Estimation Optimization (Adam), which was used to adapt the parameter learning rate (Kingma and Ba, 2015). A dropout rate of 0.3 was applied in order to reduce overfitting and improve model performance. The batch size and epoch number were set as 128 and 20, respectively. The RNN was built using the Keras API in Python with a TensorFlow (version 1.14) backend, and in the development environment of Jupyter Notebook.

Class imbalance is a well-known problem that can result in a classification bias toward the majority class (Japkowicz, 2000). Since our dataset was drawn from participants naturally performing activities, the training set of samples was not balanced among various verb and TO classes (see sample sizes in **Figure 5**). An imbalance in TO classes might also result from sorting and indexing the objects as described in section Input Features for the Action Primitive Recognition Model. For instance, Object 1 occurs most frequently in the GOS by definition. Thus, Object 1 is more likely to be the target object than Objects 2 or 3. In order to compensate for the class imbalance, each class' contribution in the cross-entropy loss function was weighted by its corresponding number of samples (Aurelio et al., 2019).

The temporal sequence of the target object and verb recognized by the RNN can contain abrupt changes, as shown in the top rows of **Figures 5A,B**. These abrupt changes occur for limited time instances and make the continuous model prediction unsmooth. Such unstable classifier results might cause an assistive robot to respond unexpectedly. Thus, we implemented a one-dimensional mode filter with an order of m (in our work, *m* = 12 time steps, equivalent to 0.2 s) to smooth out these sequences (Wells, 1979):

(6) $$\begin{array}{r} \text{verb}\left(t_{i}\right)=\text{mode}\left(\left\{verb\left(t_{i-m}\right),verb\left(t_{i-m+1}\right),\ldots ,verb\left(t_{i-1}\right)\right\}\right) \end{array}$$

(7) $$\begin{array}{r} \text{ TO}\left(t_{i}\right)=\text{mode}\left(\left\{TO\left(t_{i-m}\right),TO\left(t_{i-m+1}\right),\ldots ,TO\left(t_{i-1}\right)\right\}\right) \end{array}$$

The sequences after filtering are shown in the middle rows of **Figures 5A,B**.

Considering that 10 subjects participated in our study, we adopted a leave-one-out cross-validation method. That is, when one subject's data were reserved for testing, the other nine subjects' data were used for training.

## Performance Metrics for Action Recognition

In order to evaluate the performance of the action primitive classification, we assessed overall accuracy, precision, recall, and the F1-score. Overall accuracy is the number of correctly classified samples divided by the total size of the dataset. For each class of verb or target object, precision represents the fraction of correctly recognized time steps that actually belong to the given class, and recall represents the fraction of the class that are successfully recognized. We use TP, TN, and FP to represent the number of true positives, true negatives, and false positives when classifying a verb or target object class.

(8) $$\begin{array}{r} \text{overall accuracy}=\;\frac{\sum TP}{total\;size\;of\;dataset} \end{array}$$

(9) $$\begin{array}{r} \text{precision}=\frac{TP}{TP+FP} \end{array}$$

(10) $$\begin{array}{r} \text{recall}=\frac{TP}{TP+TN} \end{array}$$

The F1-score is the harmonic mean of precision and recall.

(11) $$\begin{array}{r} F_{1}=\frac{2\cdot precision\cdot recall}{precision+recall} \end{array}$$

We also used performance metrics that were related to the temporal nature of the data. In order to evaluate how early an action primitive was successfully recognized, we adopted the terminology “observational latency,” as defined in Ellis et al. (2013). The term was defined as “the difference between the time a subject begins the action and the time the classifier classifies the action,” which translates to the amount of time that a correct prediction lags behind the start of an action primitive. It should be noted that the observational latency does not include the computation time that the recognition algorithm requires to preprocess the input data and recognize the actions by the model.

We conservatively judged the success of an action primitive's classification by checking whether more than 75% of its time period was predicted correctly. Summary statistics for observational latency are reported for action primitives that were deemed correct according to this 75% threshold. Observational latency is negative if the action primitive is predicted before it actually begins.

## Results

Recall our aim of specifying the three components of the action primitive triplet: verb, target object, and hand object. Given that the hand object is already known, as described in section Input Features for the Action Primitive Recognition Model, we report on the ability of the RNN to recognize the verb and target object. A demonstration of the trained RNN is included in Supplementary Video 1.

### Effect of Time Window Size on Recognition Accuracy

**Figure 4:**
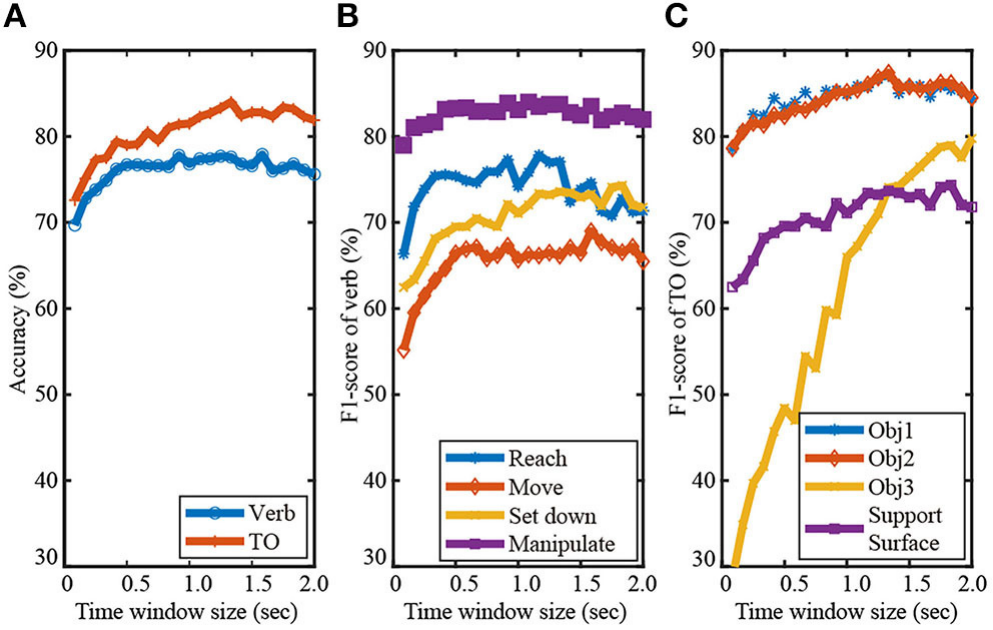
The effect of time window size (ranging from 83 ms to 2 s) on recognition performance is shown for Activity 1. The overall recognition accuracy for verb and target object are shown in **(A)**. F1-scores for the verb and target object classes are shown in **(B,C)**, respectively.

In order to set the time window size, we conducted a pilot study inspired by Haseeb and Parasuraman (2017). We tested how the F1-scores of the verb and TO classes varied as the time window size was increased from five time steps (equivalent to 83 ms) to 2 s in increments of five time steps (Figure 4). Considering the average duration of an action primitive was only 1.2 s, we did not consider time window sizes beyond 2 s.

As seen in Figure 4A, time window size had a more substantial effect on the recognition of TO than that of verb. This is due to the fact that time window size can greatly affect the data sample distributions among target object classes as a result of sorting and indexing the activity-relevant objects. Figure 4C shows that the TO class Object 3 was especially sensitive to the window size. The corresponding F1-score continuously increased from ~30% to 80% until the window size reached 1.8 s. Recognition performance of the other three TO classes Object 1, Object 2, and Support surface were also improved as the time-window size was increased from 80 ms to 1.25 s. The increased F1-scores of the TO classes can be partly attributed to alleviated class imbalance problem as the time window was lengthened, especially for the class Object 3. The number of data samples of Object 3 greatly increased due to the nature of sorting and indexing objects according to their frequency of occurrence in gaze object sequence.

As seen in Figure 4B, the F1-scores of the verb classes Reach, Move, and Manipulate increased as the time-window size increased from 80 ms to 0.5 s. Little improvement in the F1-scores was observed for time window sizes > 0.5 s, except for Set down. This suggested that a memory buffer of 0.5 s might be sufficient for predicting the verb class based on eye gaze. Gaze-related information collected long before the start of an action primitive was very likely to be irrelevant to the verb.

Considering the effect of the time window size on the classification accuracy of both the verb and target object (Figure 4), we decided to use a time window size of 1.25 s. A time window longer than 1.25 s might slightly improve recognition performance, but with additional computational cost.

### Intra-Activity Recognition

**Figure 5:**
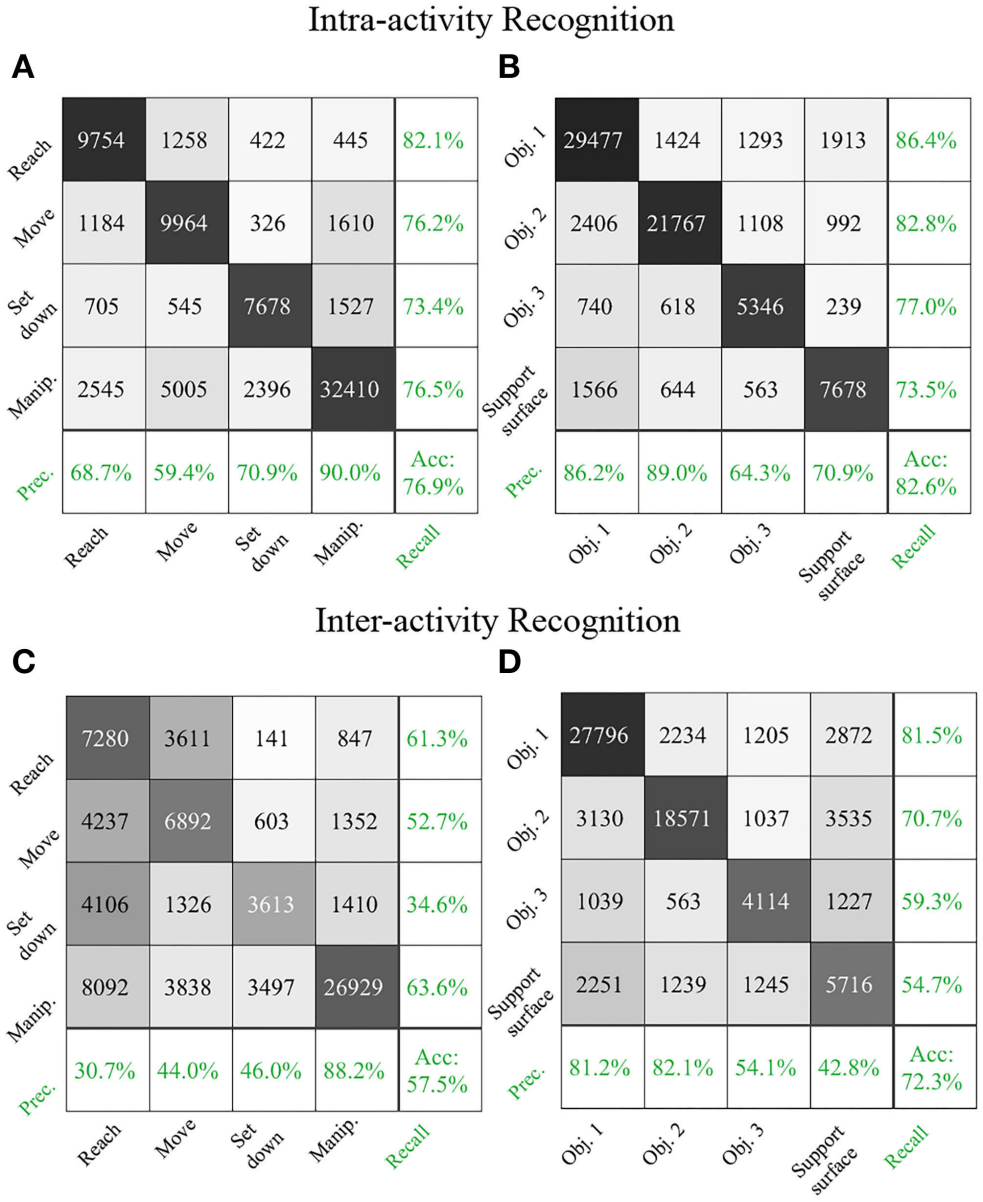
Intra-activity recognition results for Activity 1 are shown in confusion matrix form for **(A)** verb and **(B)** target object. Inter-activity recognition results for an RNN trained on Activity 2 and tested on Activity 1 are shown for **(C)** verb and **(D)** target object. Integers in the confusion matrices represent numbers of samples. The confusion matrices are augmented with precision, recall, and accuracy results (green).

We report results for intra-activity recognition, in which we trained and tested the recurrent neural network on the same activity. These results describe how well the RNN recognized novel instances of each activity despite variability inherent to activity repetition. Intra-activity recognition results for Activity 1 are shown in Figure 5 in the traditional form of confusion matrices. The rows correspond to the true class and the columns correspond to the predicted class. For brevity, intra-activity recognition results for Activities 1 and 2 are also shown in Table 2 in the form of F1-scores. The weighted averages of F1-scores for verb and target object were each calculated by taking into account the number of data samples for each class. The RNN was not trained on Activity 3 due to its smaller dataset as compared to Activities 1 and 2. Thus, no intra-activity recognition results were reported for Activity 3.

**Table 2:** The RNN performance for intra- and inter-activity recognition is reported via F1-scores (%). Weighted averages of F1-scores that account for the number of data samples in each class are reported for both verb and target object (TO).

**Intra- or Inter-activity recognition**	**Intra**	**Inter**	**Inter**	**Intra**	**Inter**	**Inter**
Activity # (training)	1	1	1	2	2	2
Activity # (testing)	1	2	3	2	1	3
**F1-scores for verb recognition (%)**						
Reach	74.8	52.9	54.8	56.5	40.9	55.6
Move	66.8	36.6	61.1	59.5	48.0	60.5
Set down	72.1	49.3	45.3	59.6	39.5	44.4
Manipulate	82.7	73.7	72.7	81.4	73.9	71.8
*Verb Average*	77.4	60.3	63.6	68.6	59.9	63.1
**F1-scores for target object recognition (%)**						
Object 1	86.3	72.1	78.0	80.2	81.3	77.4
Object 2	85.8	80.7	83.6	87.2	76.0	80.8
Object 3	70.1	41.7	52.5	55.2	56.6	56.8
Support surface	72.2	56.9	49.8	69.3	48.0	46.6
*TO Average*	82.8	73.0	74.9	81.1	72.8	73.4

We augmented the traditional confusion matrix used to report results according to true and predicted classes with additional metrics of precision and recall (Figure 5). Precision and recall were reported as percentages (in green) in the far right column and bottom-most row, respectively. The cell in the lower-right corner represented the overall recognition accuracy.

The data samples were not balanced among various verb and TO classes since our dataset was drawn from participants naturally performing activities. The proportion of each verb and TO class in Activity 1 was the sum of the corresponding row in Figures 5A,B divided by the total size of the dataset (77,774 time step samples). The proportions for the verb classes were 15% for Reach, 17% for Move, 13% for Set down, and 55% for Manipulate. The proportions for the target object classes were 44% for Object 1, 34% for Object 2, 9% for Object 3, and 13% for Support surface.

The RNN achieved a good performance in recognizing the majority verb class Manipulate (precision: 90%, recall: 77%) and the TO class Object 1 (precision: 86%, recall: 86%), which laid a solid foundation for its overall accuracy (verb: 77%, TO: 83%).

### Inter-activity Recognition

We report results for inter-activity recognition, in which we trained and tested the recurrent neural network on different activities. These results describe how well the RNN can recognize verbs and target objects despite variability across different activities. To evaluate the algorithm's cross-activity generalizability, an RNN trained on Activity 2 (make instant coffee) was tested on Activity 1 (make a powdered drink), and vice versa. RNNs trained on Activity 1 and Activity 2 were additionally tested on Activity 3 (prepare a cleaning sponge). The confusion matrices of an RNN trained on Activity 2 and tested on Activity 1 are shown in Figures 5C,D for verb and target object estimation, respectively. For brevity, additional inter-activity recognition results are presented in Table 2 in the form of F1 scores.

We also compared intra-activity and inter-activity performance of RNN models tested on the same activity. For this, we subtracted the average F1-scores for inter-activity recognition from those of the appropriate intra-activity recognition for RNNs tested on Activity 1 and Activity 2. As expected, when testing with an activity that differed from the activity on which the RNN was trained, the classification performance decreased. The average F1-scores of verb and target object each dropped by 8% when the RNN was trained on Activity 1 and tested on Activity 2. The average F1-scores of verb and target object dropped by 18 and 10%, respectively, when the RNN was trained on Activity 2 and tested on Activity 1. The average F1-score decreases were no larger than 20%, which suggested that the classification algorithm was able to generalize across activities to some degree. In addition, despite the fact that Activity 3 shared only one common activity-relevant object (mug) with the other two activities, the average F1-scores of verb and TO achieved for Activity 3 were slightly higher than those of the other inter-activity recognition tests (Table 2).

### Effect of Input Features on Recognition Accuracy

**Figure 6:**
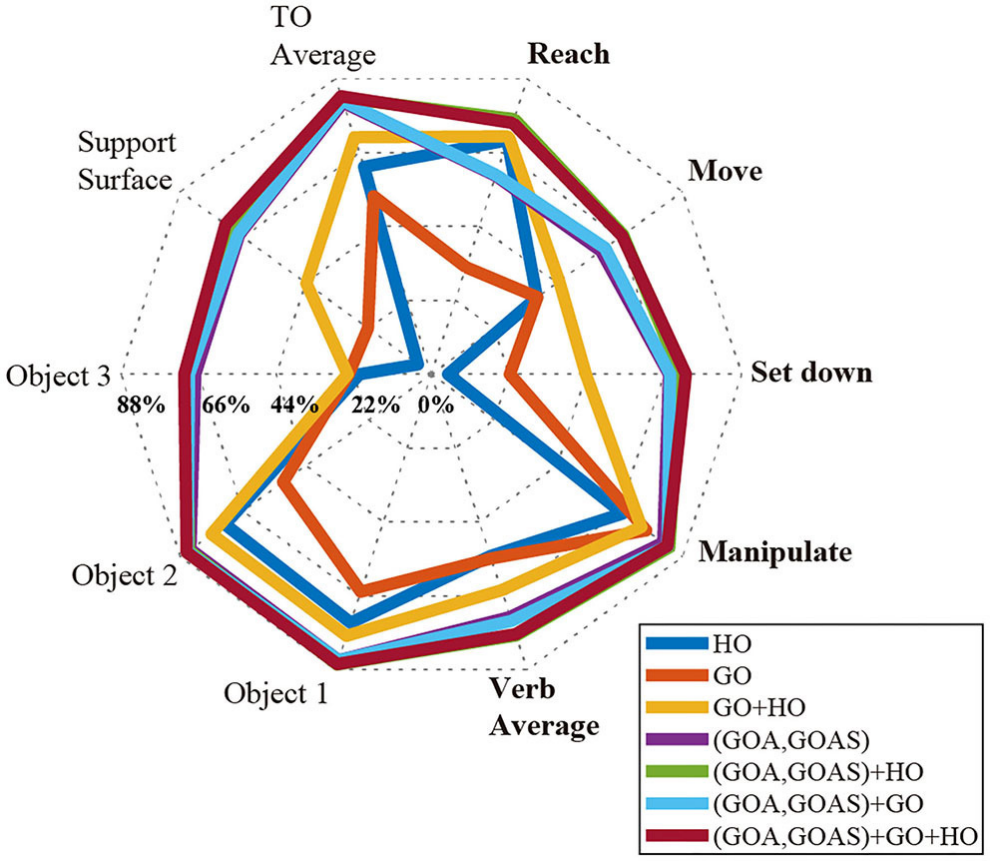
For Activity 1, RNN performance is reported by F1-scores for different combinations of input features (HO, GO, GOA, GOAS) using a radar chart. Axes represent the verb (bold) and target object classes. F1-score gridlines are offset by 22%. Each of the polygons corresponds to one combination of input features. The combined use of HO, GO, GOA, and GOAS features resulted in the best performance; HO alone performed the worst.

In order to evaluate feature importance, we compared the classification performance achieved in Activity 1 with various combinations of input features using a radar chart (Figure 6). Axes represented the verb and target object classes. Gridlines marked F1-scores in increments of 22%. Classification using HO alone was poor, with F1-scores for “Set down” and “Object 3” being <10%. Only slightly better, classification using GO alone was still not effective, with F1-scores of the “Set down,” “Object 3,” and “Support surface” only reaching values near 22%. In contrast, GOA-based features (GOA, GOAS) alone outperformed both HO and GO on their own in every verb and target object class. With the exception of “Reach,” GOA-based features alone also outperformed the use of HO and GO together.

Although the feature HO alone did not provide good recognition result, it could substantially improve the classification performance when used in concert with GOA-based features. For every class, the F1-scores achieved with the combination of GOA-based feature and HO were equal to or higher than with the GOA-based feature alone.

### Effect of Input Features on Observational Latency

**Figure 7:**
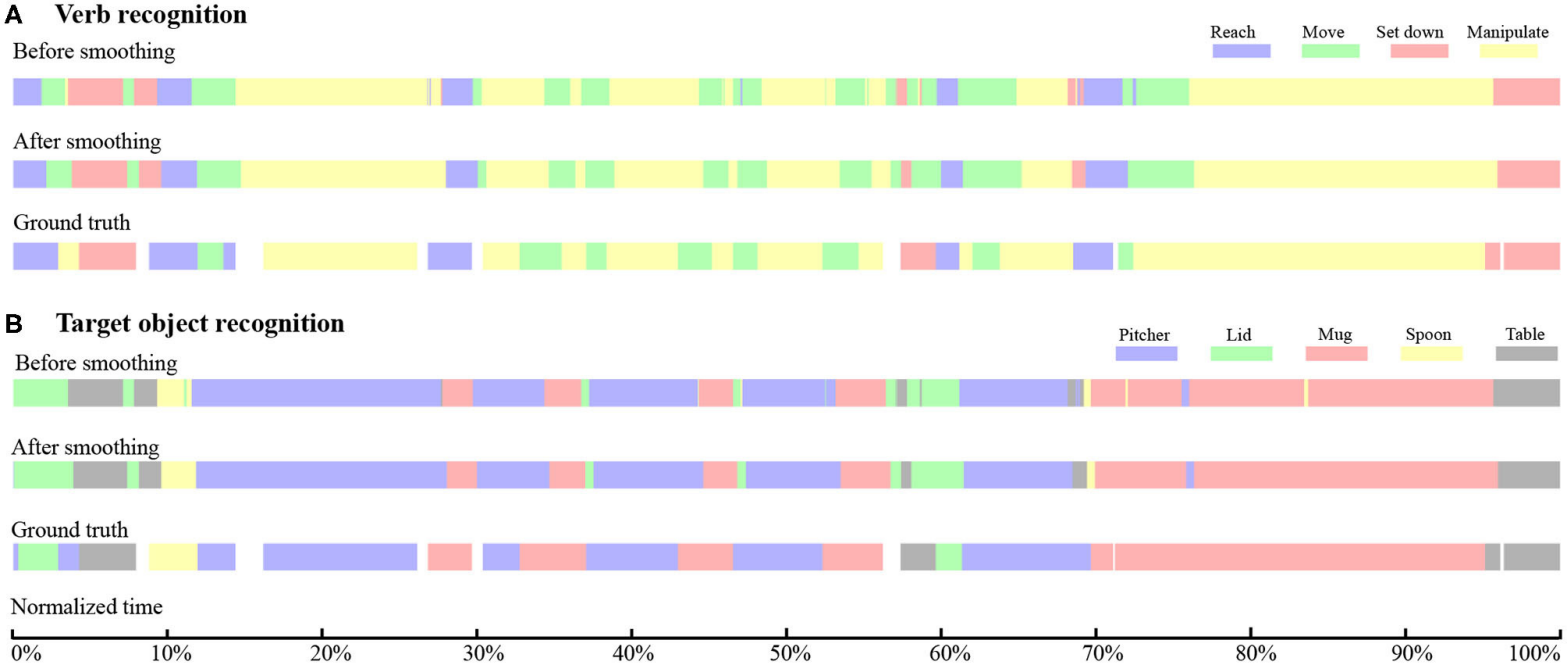
For a representative trial of Activity 1, temporal sequences of recognition results and ground truth are presented for **(A)** verb and **(B)** target object. In both **(A,B)**, the top, middle, and bottom color bars represent the raw RNN output, RNN output smoothed by a mode filter, and hand-labeled ground truth, respectively. The total duration of this trial is 36 s.

The time histories of the verb and target object recognition for a representative Activity 1 trial are shown in Figures 7A,B. In each of Figures 7A,B, the top colorbar represents a time history of raw prediction results. The middle colorbar shows the output of the mode filter that smooths the raw prediction results. The bottom colorbar represents the ground truth. White gaps in the ground truth correspond to instances when neither hand was moving or manipulating an object. The observational latency is obtained by comparing the middle and bottom colorbars.

**Figure 8:**
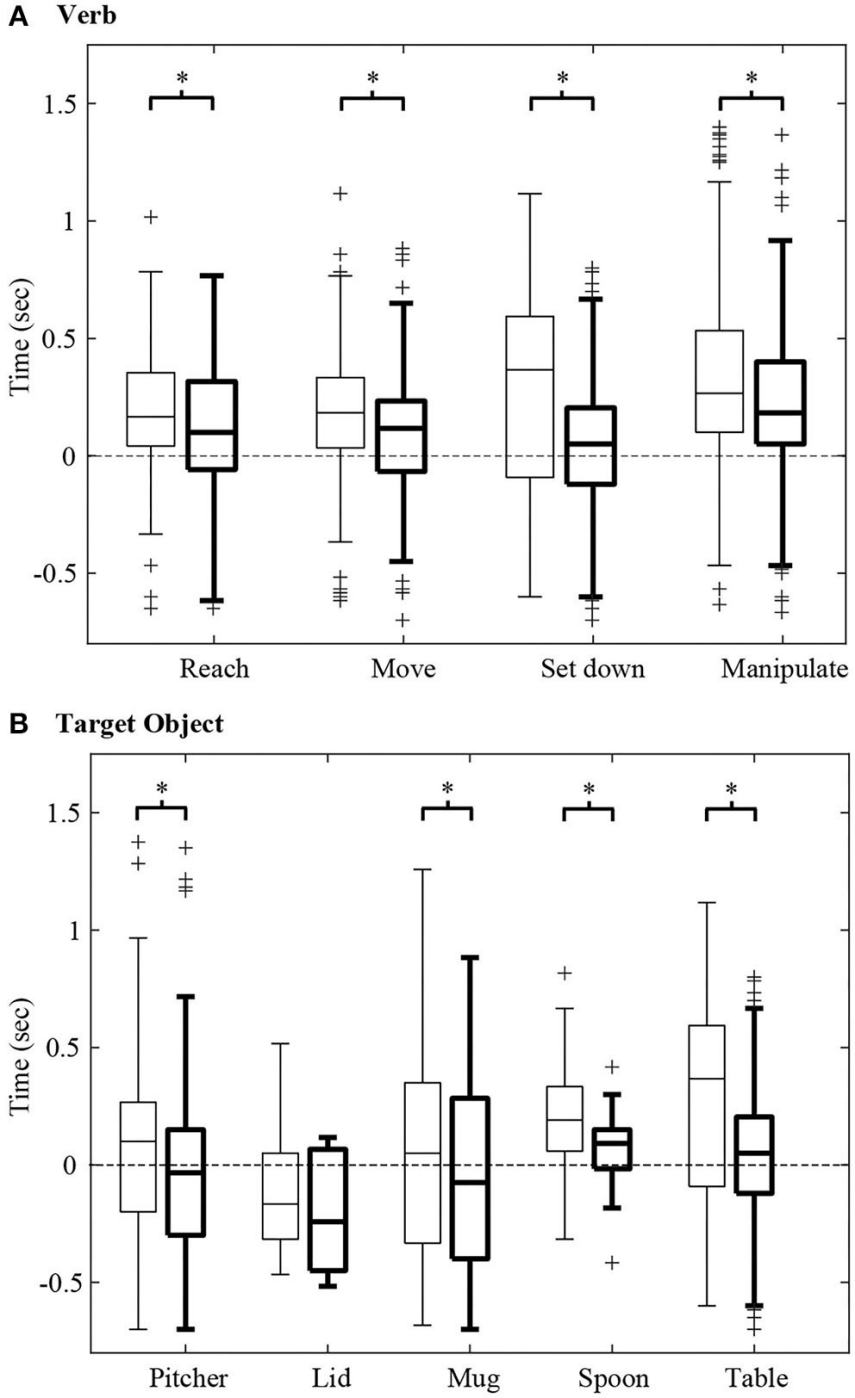
For Activity 1, the observational latency for recognition of **(A)** verb and **(B)** target object are shown using box and whisker plots. A negative latency value indicates that a verb or target object is identified before the start of the action primitive. For each boxplot pair, the observational latency without using GOA and GOAS (thin lines) is compared with that using GOA and GOAS (thick lines). Each boxplot indicates the 25, 50, and 75th percentiles. The whiskers extend to the most extreme data points that are not considered outliers (“+”) having values of more than 1.5 times the interquartile range from the top or bottom of the box. Asterisks indicate *p* < α = 0.05.

While Figure 7 shows the observational latency for a single representative trial, the observational latencies for all trials and participants are presented in Figure 8. Specifically, Figures 8A,B, summarize results for the recognition of verb and target object, respectively, for an RNN trained and tested on Activity 1. Figure 8 illustrates the effect of input features on observational latency by comparing the results of an RNN that only used GO and HO as input features to those of an RNN that additionally used GOA, and GOAS as input features.

We hypothesized that the incorporation of GOA-based input features could significantly decrease observational latency. To test this, we conducted a Wilcoxon signed-rank test (following a Lilliefors test for normality) with a total of 714 action primitives. The one-tailed *p*-values for the verbs and target objects were all less than the α level of 0.05 except for the target object of pitcher lid. Thus, we concluded that the use of GOA and GOAS as input features in addition to GO and HO resulted in a reduction in observational latency (Figure 8).

## Discussion

### Features Based on Gaze Object Angle Improve Action Primitive Recognition Accuracy

The long-term objective of this work is to advance shared autonomy control schemes so that individuals with upper limb impairment can more naturally control robots that assist with activities of daily living. One embodiment of such a teleoperated system could include both a joystick and eyetracker as user input devices. The short-term goal of this study was to improve action primitive recognition accuracy and observational latency. We pursued this goal by (i) focusing on the recognition of low-level action primitives, and (ii) defining eye gaze-based input features that improve action primitive recognition.

Previous studies leveraged egocentric videos to recognize actions when a subject was naturally performing ADLs. The features reported in these studies can be divided into three categories: features based on human hands, objects, or human gaze. Examples of hand-based features include hand location, hand pose, and relative location between left and right hands (Fathi et al., 2011; Ma et al., 2016). Fathi et al. relied on changes in the state of objects, such as the state of the “coffee jar” (open vs. closed) (Fathi and Rehg, 2013), to recognize actions. Behera et al. used spatiotemporal relationships between objects as classifier inputs (Behera et al., 2012). Features related to human gaze included the gaze-object, which was widely used to classify actions (Yi and Ballard, 2009; Matsuo et al., 2014). The use of object appearance (histogram of color and texture) in the neighborhood of the gaze point was also effective in improving recognition accuracy (Fathi et al., 2012; Li et al., 2015b).

Considering the long-term objective of this work, we elected not to rely solely on features based on human hands or objects for action primitive recognition. Features based on human hands are only available when subjects use their own hands to directly grasp and manipulate objects. For the assistive robot application we envision, features of human hands such as hand location, hand pose, and relative location between left and right hands (Fathi et al., 2011; Ma et al., 2016) will not be available. Features based on objects are consequence of hand motions, such as changes in the states of objects or spatiotemporal relationships between objects. Such object-based features would only be available in hindsight and cannot be collected early enough to be useful for the proposed assistive robot application.

We aim to exploit observations that gaze behavior is a critical component of sighted grasp and manipulation activities, and that eye movements precede hand movements (Johansson et al., 2001; Land, 2006). In particular, it has been reported that eye gaze often shifts to a target object before any hand movement is observed (Land and Hayhoe, 2001). As such, we adopted the gaze-based feature GO from the literature (e.g., Yi and Ballard, 2009) and supplemented it with two new features that we defined: GOA and GOAS.

As reported in section Effect of Input Features on Recognition Accuracy, models that included GOA and GOAS as input features outperformed models that relied primarily on GO or HO for every verb and target object class. The addition of GOA and GOAS substantially improved the average F1-score from 64% to 77% for verb and from 71 to 83% for target object (Figure 6).

The advantages of using features based on gaze object angle for action primitive recognition are 2-fold. First, the gaze object angle quantifies the spatiotemporal relationship between the gaze vector and every object in the workspace, including objects upon which the subject is not currently gazing. In contrast, the gaze object only captures the identity of the object upon which the subject is gazing at that particular instant. Considering that daily activities generally involve a variety of objects, it is vital for the classifier to collect sufficient information related to gaze-object interactions. The feature GOA could indirectly provide information similar to that of GO. For example, a GOA value that is close to zero would result if the gaze vector is essentially pointing at the gaze object. When GOA, GOAS, and HO have already been included as input features, the addition of GO as an input feature has little to no impact on classification accuracy (Figure 6). Also, classifier performance improves when using GOA and GOAS as input features as compared to using GO, HO, or their combination (Figure 6).

Second, the input feature GOAS contains GOA rate information. To some extent, GOAS also captures directional information, as positive and negative GOAS values reflect whether the gaze vector is approaching or departing from each object in the workspace, respectively. We believe that approach/departure information can be leveraged to predict the target object for a given action primitive because gaze is used to gather visual information for planning before and during manual activities (Land, 2006). An object being approached by the gaze vector is not necessarily the target object, as the object could simply be in the path of the gaze vector during its movement. However, objects are less likely to be labeled as the “target object” when the gaze vector moves away from them.

### Features Based on Gaze Object Angle Improve Observational Latency

While recognition accuracy is important, human-robot systems also require low observational latency (Ellis et al., 2013). Even an action primitive that is correctly recognized 100% of the time will cease to be useful if the delay in recognition prohibits an effective response or adds to the cognitive burden of the operator. The earlier that a robotic system can infer the intent of the human operator or collaborator, the more time will be available for computation and the planning of appropriate robot movements.

Previous studies have focused on classifying actions in videos that have already been segmented in time (e.g., Fathi et al., 2012). However, these methods that were designed to recognize actions in hindsight would be less effective for real-time use. We desire the intended action primitive to be predicted in advance of robot movement and with as low an observational latency as possible.

Hoffman proposed several metrics to evaluate fluency in human-robot collaborative tasks. For instance, the robot's functional delay was defined as the amount of time that the human spent waiting for the robot (Hoffman, 2019). This concept of fluency reflects how promptly a robot can respond correctly to an operator's commands. A high observational latency will degrade the fluency of a human-robot system and increase the operator's cognitive burden, effort, and frustration levels. A user interface that requires operators to intentionally gaze at specific objects or regions for a fixed period of time may be less natural and have lower fluency than a user interface that leverages natural eye gaze behaviors (Li et al., 2017; Wang et al., 2018).

In this work, the use of gaze-related features enabled the recognition of action primitives at an early stage. The average observational latency for verb recognition was 120 ms, ~10% of the average duration of an action primitive (1.2 s). The average observational latency for target object was −50 ms; the negative latency value indicates that the target object was sometimes identified before the start of the action primitive. Unfortunately, pooled across all classes, the observational latency for the target object was not statistically significantly less than zero (*p* = 0.075; α = 0.05). Nonetheless, the fact that some of the trials resulted in negative observational latency values was surprising and encouraging.

Among gaze-related input features, the use of GOA and GOAS decreased the observational latency as compared with using GO alone (Figure 8). Per a Wilcoxon signed rank test, observational latency was statistically significantly smaller when GOA and GOAS were used as input features than when they were excluded (*p* < α = 0.05). This was true for all verb classes and all target object classes, with the exception of lid. For the verb and target object, the observational latency dropped by an average of 108 and 112 ms, respectively. One reason for this could be that GOA-based features may encode the tendency of the gaze vector to approach an object once the eyes start to move. In contrast, the GO feature does not capture the identity of any object until the gaze vector reaches the object.

The sub-second observational latency values that we report likely resulted from the fact that eye movement generally precedes hand movement for manual activities (Johansson et al., 2001; Land, 2006). Land et al. reported that the gaze vector typically reached the next target object before any visible signs of hand movement during the activity of making tea (Land and Hayhoe, 2001). The small observational latency values may also result from the fact that our classifier was designed to recognize action primitives, which are much simpler than actions or activities (Moeslund et al., 2006). Action primitives often involve a single object, a single hand, and occur over a shorter period of time than actions and activities. The recognition of actions and activities for ADLs would require observations over a longer period of time and would necessarily involve more complex eye behaviors, more complex body movements, and gaze interactions with multiple objects.

Ryoo predicted activities of daily living and defined the “observation ratio” as the ratio between the observational latency and the activity duration (Ryoo, 2011). Ryoo reported that a minimum observation ratio of ~45% was needed to classify activities with at least 60% accuracy. In this work, we found that minimum observation ratios of 18 and 5% were needed to achieve an accuracy of 60% for each the verb and the target object, respectively. This suggests that recognition of low-level action primitives can be achieved at lower observation ratios and within shorter time periods than high-level activities, which require the passage of more time and collection of more information for similar levels of accuracy.

One limitation of this work is that the action primitive recognition algorithm has not yet been tested in real-time. This is an area of future work and considerations for real-time implementation are discussed in section Comparisons to State-of-the-Art Recognition Algorithms. Based on our experience, we expect that the overall latency will be dominated by observational latency and less affected by computational latency. This is due to the relatively simple structure of the proposed RNN architecture and the fact that the RNN model would be trained offline a priori.

### Segmenting Objects Into Regions According to Affordance Could Improve Recognition Performance

The distribution of gaze fixations can be concentrated on certain regions of an object, such as those associated with “object affordances.” An object affordance describes actions that could be performed on an object (Gibson, 1977). For example, Belardinelli et al. showed human subjects a 2D image of a teapot and instructed them to consider lifting, opening, or classifying the teapot as an object that could or could not hold fluid (Belardinelli et al., 2015). It was observed that subjects' gaze fixations were focused on the teapot handle, lid, and spout for lifting, opening, and classifying, respectively. In addition, in a prior study, we reported 3D gaze heat maps for the activity “make a powdered drink” (Haji Fathaliyan et al., 2018). We observed that gaze fixations were focused on the top and bottom of pitcher during the action unit “reach for pitcher” and “set down pitcher.”

**Figure 9:**
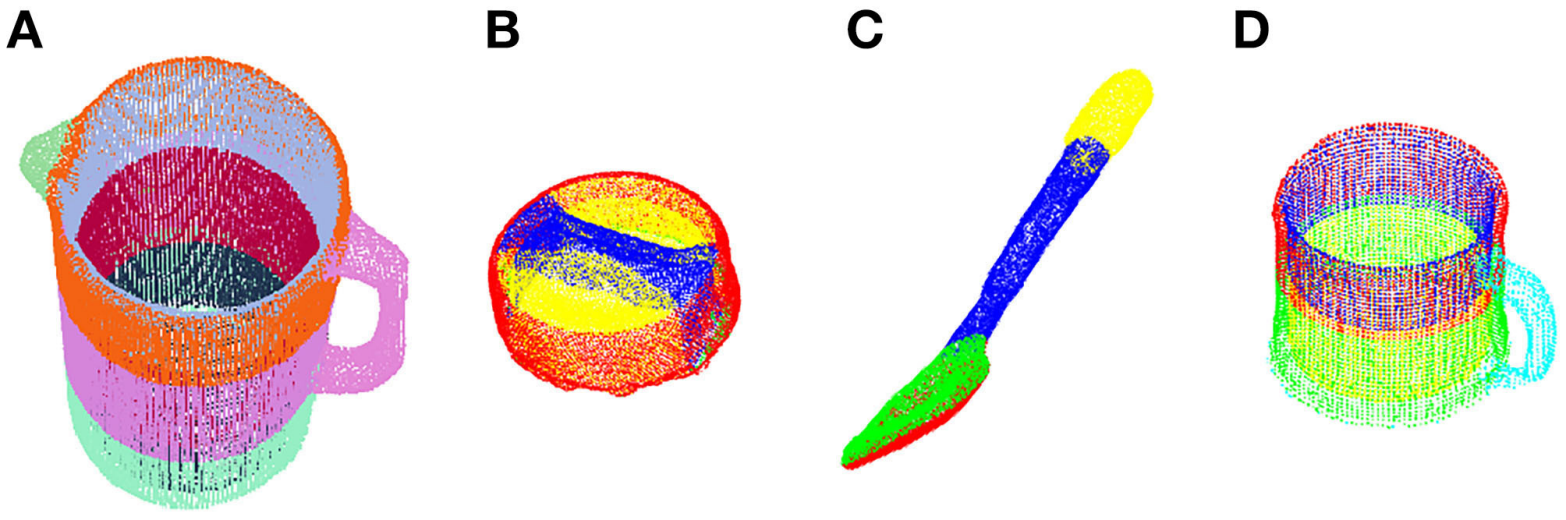
Point clouds of the four activity-relevant objects involved in Activity 1 were segmented into multiple regions for finer spatial resolution: **(A)** pitcher, **(B)** pitcher lid, **(C)** spoon, and **(D)** mug.

Inspired by these findings, we hypothesized that information about the action primitive can, in theory, be encoded by gaze behavior with respect to specific regions of objects. This would provide a classification algorithm with information at a finer spatial resolution than when considering each object as a whole. In a *post hoc* study, we segmented the point clouds of each of the four activity-relevant objects in Activity 1 (make a powdered drink) into several regions according to object affordances (Figure 9). For instance, the spoon was segmented into the upper and bottom faces for the bowl, the handle, and the tip of the handle. Notably, the inner and outer wall of containers (pitcher and mug) were treated as different regions since the inner and outer walls were often fixated upon differently depending on the action primitive.

After the segmentation, we augmented the gaze-related features (GO, GOA, GOAS) by treating each region as an independent object while keeping the features left-hand object and right-hand object unchanged. We then retrained the RNN with the new augmented features. The recognition accuracy for verb increased slightly from 77 to 79% and accuracy for the target object increased from 83 to 86%. By increasing the total number of object regions from 4 to 20, the time taken for the trained RNN to produce one classifier output increased by 26%. Depending on the consequences of an incorrect classification and the minimum acceptable accuracy level, one could decide which objects to segment and how finely the objects should be segmented. For instance, one may still be able to improve recognition performance if the mug were segmented into inner wall, outer wall, and handle, as opposed to the five segments that we tested.

### Comparisons to State-of-the-Art Recognition Algorithms

In the evaluation of our proposed gaze-based action primitive recognition method, we were unable to identify suitable benchmarks for a direct quantitative comparison. First, our approach is designed to recognize low-level action primitives that could be used as modular, generalizable building blocks for more complex levels of the action hierarchy (Moeslund et al., 2006). The literature on action recognition provides methods for recognition at the level of actions and activities, but not at the level of action primitives that are investigated in our work. For instance, the public dataset “GTEA+” and “EGTEA Gaze+” provided by Fathi et al. (2012) Li et al. (2018) involve actions such as “take bread.” This action would need to be split into two separate action primitives: “reach bread,” and “set down bread onto table.” Likewise, the public dataset “CMU-MMAC” provided by De la Torre et al. (2009) involves actions such as “stir egg.” This action would need to be split into three action primitives: “reach fork,” “move fork into bowl,” and “stir egg in the bowl using fork.” Many state-of-the-art recognition methods for ADLs (whether leveraging gaze behavior or not) are based on these publicly available datasets at the action level.

Second, action recognition models in the literature rely on computer-vision based approaches to analyze 2D videos recorded by an egocentric camera, e.g., (Fathi et al., 2011, 2012; Fathi and Rehg, 2013; Matsuo et al., 2014; Soran et al., 2015; Ma et al., 2016; Li et al., 2018; Furnari and Farinella, 2019; Sudhakaran et al., 2019; Liu et al., 2020). Whether using hand-crafted features (Fathi et al., 2011, 2012; Fathi and Rehg, 2013; Matsuo et al., 2014; Soran et al., 2015; Ma et al., 2016; Furnari and Farinella, 2019) or learning end-to-end models (Li et al., 2018; Sudhakaran et al., 2019; Liu et al., 2020), the computer vision-based approaches to action recognition must also address the challenges of identifying and tracking activity-relevant objects. In contrast, we bypassed the challenges inherent in 2D image analysis by combining an eyetracker with a marker-based motion capture system. This experimental set-up enabled the direct collection of 3D gaze-based features and object identity and pose information so that we could focus on the utility of 3D gaze features, which are unattainable from 2D camera images. Our method could be introduced into non-lab environments by combining an eyetracker with 2D cameras and ArUco markers, for example, in place of a marker-based motion capture system.

### Considerations for Real-Time Implementation of an Action Primitive Recognition Algorithm in Human-Robot Systems

As an example of how our action primitive recognition model could be applied in a human-robot shared autonomy scenario, consider the action “stir contents inside a mug.” First, as a subject's eye gaze vector moves toward the spoon, the probability of the potential action primitive “reach spoon” increases until it exceeds a custom threshold. The crossing of the threshold triggers the robotic end effector to move autonomously toward the spoon handle in order to grasp the spoon. The robot would use its real-time 3D model of the scene to plan its low-level movements in order to reduce the cognitive burden on the human operator. Second, as the subject's eye gaze switches to the mug after a successful grasp of the spoon, the model would recognize the highest probability action primitive as “move spoon to mug.” Again the crossing of a probability threshold, or confidence level, would trigger the autonomous placement of the grasped spoon within the mug for a subsequent, allowable manipulate-type action primitive, which would be limited to a set of allowable manipulate-type action primitives based on the gaze object and hand object. Third, as the subject fixates their gaze on the mug, the model would recognize the highest probability action primitive as “stir inside mug” and autonomous stirring would begin. The stirring trajectory could be generated using parametric dynamic motion primitives (Schaal, 2006), for example. Lastly, as the subject's gaze saccades to a support surface and the action primitive is recognized as “set down spoon,” the system would proceed to determine a location on the table at which to place the spoon. This exact location could be extracted from filtered eye gaze signals as introduced in Li et al. (2015a).

As described in the above example, we envision that our model could be used to recognize subjects' intended action primitives through their natural eye gaze movements while the robot handles the planning and control details necessary for implementation. In contrast to some state-of-the-art approaches to commanding robot movements (Li and Zhang, 2017; Wang et al., 2018; Shafti et al., 2019; Zeng et al., 2020), subjects would not be forced to unnaturally, intentionally fixate their gaze at target objects in order to trigger pre-programmed actions. Of course, much work is necessary to implement the proposed shared autonomy control scheme and this is the subject of future work.

Concerning the practical implementation of the proposed action primitive recognition method, several limitations must be addressed.

#### Specificity of the Action Primitive

The proposed recognition method is intended to assign generalized labels to each time step as one of the four verb classes (reach, move, set down, and manipulate). The current method does not distinguish between subclasses of manipulate-type verbs, such as “pour” and “stir.” Recognition of subclasses of a verb could enable assistive robots to provide even more specific assistance than that demonstrated in this work.

Recognition specificity could be advanced by incorporating additional steps. One idea is to create a lookup table based on the affordances of the objects involved in the activities. For example, the action primitive triplet of (verb = manipulate, TO = mug, HO = pitcher) is associated with the verb subclass “pour.” However, the triplet (verb = manipulate, TO = pitcher, HO = spoon) is associated with both verb subclasses “stir” and “scoop.” As an alternative, we suggest the use of gaze heat maps to facilitate the classification of verb subclasses since action primitives are activity-driven and the distribution of gaze fixations can be considerably affected by object affordance (Belardinelli et al., 2015; Haji Fathaliyan et al., 2018).

#### Distracted or Idle Eye Gaze States

The proposed recognition method does not recognize human subjects' distracted or idle states. For example, a subject's visual attention can be distracted by environmental stimuli. In this study, we minimized visual distractions through the use of black curtains and by limiting the objects in the workspace to those required for the instructed activity. The incorporation of distractions (audio, visual, cognitive, etc.) is beyond the scope of this work, but would need to be addressed before transitioning the proposed recognition method to natural, unstructured environments.

Idle states are not currently addressed in this work. Hands are not used for every activity and subjects may also wish to rest. If the gaze vector of a daydreaming or resting subject happens to intersect with an activity-relevant object, an assistive robot may incorrectly recognize an unintended action primitive and perform unintended movements. This is similar to the “Midas touch” problem in the field of human-computer interaction, which faces a similar challenge of “how to differentiate ‘attentive' saccades with intended goal of communication from the lower level eye movements that are just random” (Velichkovsky et al., 1997). This problem can be addressed by incorporating additional human input mechanisms, such as a joystick, which can be programmed to reflect the operator's agreement or disagreement with the robot's movements. The inclusion of “distracted” and “idle” verb classes would be an interesting area for future advancement.

#### Integration With Active Perception Approaches

The proposed recognition method could be combined with active perception approaches that could benefit a closed-loop human-robot system that leverages the active gaze of both humans and robots. In this work, the 3rd person cameras comprising the motion capture system passively observed the scene. However, by leveraging the concept of “joint attention” (Huang and Thomaz, 2010), one could use an external and/or robot-mounted camera set-up to actively explore a scene and track objects of interest, which could be used to improve the control of a robot in a human-robot system.

As discussed in section Comparisons to State-of-the-Art Recognition Algorithms, for the purposes of this work, we bypassed the process of identifying and locating activity-relevant objects by implementing a marker-based motion capture system in our experiment. Nonetheless, the perception of activity-relevant objects in non-laboratory environments remains a challenge due to object occlusions and limited field of view. Active perception-based approaches could be leveraged in such situations. In multi-object settings, such as a kitchen table cluttered with numerous objects, physical camera configurations could be actively controlled to change 3rd person perspectives and more accurately identify objects and estimate their poses (Eidenberger and Scharinger, 2010). Once multiple objects' poses are determined, a camera's viewpoint could then be guided by a human subject's gaze vector to reflect the subject's localized visual attention. Since humans tend to align visual targets with the centers of their visual fields (Kim et al., 2004), one could use natural human gaze behaviors to control camera perspectives (external or robot-mounted) in order to keep a target object, such as one recognized by our proposed recognition method, in the center of the image plane for more stable computer vision-based analysis and robotic intervention (Li et al., 2015a). When realized by a visible robot-mounted camera, the resulting bio-inspired centering of a target object may also serve as an implicit communication channel that provides feedback to a human collaborator. Going further, the camera's perspective could be controlled actively and autonomously to focus on the affordances of a target object after a verb-TO pair is identified using our proposed recognition method. Rather than changing the physical configuration of a camera to center an affordance in the image plane, one could instead focus a robot's attention on an affordance at the image processing stage (Ognibene and Baldassare, 2015). For instance, the camera's foveal vision could be moved to a pitcher's handle in order to guide a robot's reach-to-grasp movement. Such focused robot attention, whether via physical changes in camera configuration or via digital image processing methods, could be an effective way to maximize limited computational resources. The resulting enhanced autonomy of the robot could help to reduce the cognitive burden on the human in a shared autonomy system.

Considering the goal of our work to infer human intent and advance action recognition for shared autonomy control schemes, one could also integrate our proposed methods with the concept of “active event recognition,” which uses active camera configurations to simultaneously explore a scene and infer human intent (Ognibene and Demiris, 2013). Ognibene and Demiris developed a simulated humanoid robot that actively controlled its gaze to identify human intent while observing a human executing a goal-oriented reaching action. Using an optimization-based camera control policy, the robot adjusted its gaze in order to minimize the expected uncertainty over numerous prospective target objects. It was observed that the resulting robot gaze gradually transitioned from the human subject's hand to the true target object before the subject's hand reached the object. As future work, it would be interesting to investigate whether and how the integration of 1st person human gaze information, such as that collected from an ego-centric camera, could enhance the control of robot gaze for action recognition. For instance, the outputs of our proposed action primitive recognition method (verb-TO pairs) could be used as additional inputs to an active event recognition scheme in order to improve recognition accuracy and reduce observational latency.

#### Effects of the Actor on Eye Gaze Behavior

The proposed recognition model was trained using data in which non-disabled subjects were performing activities with their own hands instead of subjects with upper-limb impairment who were observing a robot that was performing activities. In our envisioned human-robot system, we seek to identify operator intent via their natural gaze behaviors before any robotic movements occur. It is known that gaze behaviors precede and guide hand motions during natural hand-eye coordination (Hayhoe et al., 2003). In contrast, we hypothesize that the eye gaze behaviors of subjects observing robots may be reactive in nature. Aronsen et al. have shown that subjects' gaze behaviors are different in human-only manipulation tasks and human-robot shared manipulation tasks (Aronson et al., 2018). The further investigation of the effect of a robot on human eye gaze is warranted, but is beyond the scope of this work. We propose that the eye gaze behaviors reported in this work could be used as a benchmark for future studies of human-robot systems that seek to recreate the seamlessness of human behaviors.

The direct translation of the model to a human-robot system may not be possible. For one, the robot itself would need to be considered as an object in the shared workspace, as it is likely to receive some of the operator's visual attention. Fortunately, as suggested by Dragan and Srinivasa in Dragan and Srinivasa (2013), the action primitive prediction does not need to be perfect since the recognition model can be implemented with a human in the loop. The robotic system could be designed to wait until a specific confidence level for its prediction of human intent has been achieved before moving.

Another important consideration is that the recognition of action primitives via human eye gaze will necessarily be affected by how the robot is programmed to perform activities. For example, eye gaze behaviors will depend on experimental variables such as manual teleoperation vs. preprogrammed movements, lag in the robot control system and processing for semi-autonomous behaviors (e.g., object recognition), etc. Recognizing that there are innumerable ways in which shared autonomy could be implemented in a human-robot system, we purposely elected to eliminate the confounding factor of robot control from this foundational work on human eye-hand coordination.

#### Integration of Low-Level Action Primitive Recognition Models With Higher Level Recognition Models

This work focused on the recognition of low-level action primitives. However, the envisioned application to assistive robots in a shared autonomy schema would require recognition at all three hierarchical levels of human behavior (action primitives, actions, activities) (Moeslund et al., 2006) in order to customize the degree of autonomy to the operator (Kim et al., 2012; Gopinath et al., 2017). For instance, the outputs of the low-level action primitive recognition models (such as in this work) could be used as input features for the mid-level action recognition models (e.g., Haji Fathaliyan et al., 2018), that would then feed into the high-level activity recognition models (Yi and Ballard, 2009). Simultaneously, knowledge of the activity or action can be leveraged to predict lower level actions or action primitives, respectively.

## Conclusion

The long-term objective of this work is to advance shared autonomy by developing a user-interface that can recognize operator intent during activities of daily living via natural eye movements. To this end, we introduced a classifier structure for recognizing low-level action primitives that incorporates novel gaze-related features. We defined an action primitive as a triplet comprised of a verb, target object, and hand object. Using a non-specific approach to classifying and indexing objects, we observed a modest level of generalizability of the action primitive classifier across activities, including those for which the classifier was not trained. We found that the gaze object angle and its rate of change were especially useful for accurately recognizing action primitives and reducing the observational latency of the classifier. In summary, we provide a gaze-based approach for recognizing action primitives that can be used to infer the intent of a human operator for intuitive control of a robotic system. The method can be further advanced by combining classifiers across multiple levels of the action hierarchy (action primitives, actions, activities) (Moeslund et al., 2006) and finessing the approach for real-time use. We highlighted the application of assistive robots to motivate and design this study. However, our methods could be applied to other human-robot applications, such as collaborative manufacturing.

## Data Availability Statement

The datasets presented in this article are not readily available because they were not intended for public dissemination as raw data. Requests to access the datasets should be directed to Veronica J. Santos, vjsantos@ucla.edu.

## Ethics Statement

The studies involving human participants were reviewed and approved by University of California, Los Angeles Institutional Review Board. The participants provided their written informed consent to participate in this study. Written informed consent was obtained from the individual(s) for the publication of any potentially identifiable images or data included in this article.

## Author Contributions

XW and AH supervised data collection. XW performed the data analysis, interpretation, and assisted by AH. XW created the first draft of the manuscript, which was further edited by VS and AH. All authors have read and approved the submitted manuscript and contributed to the conception and design of the study.

## Conflict of Interest

The authors declare that the research was conducted in the absence of any commercial or financial relationships that could be construed as a potential conflict of interest.

## Supplementary Material

Supplementary Video 1 The Supplemental Video shows (i) a description of the experimental set-up and gaze vector reconstruction, (ii) a description of the preparation of input features for the recurrent neural network (RNN), and (iii) a demonstration of the RNN recognition of the verb and target object.
